# 6-Iodo-4-oxo-4*H*-chromene-3-carbaldehyde

**DOI:** 10.1107/S1600536814012471

**Published:** 2014-06-04

**Authors:** Yoshinobu Ishikawa

**Affiliations:** a School of Pharmaceutical Sciences, University of Shizuoka, 52-1 Yada, Suruga-ku, Shizuoka 422-8526, Japan

## Abstract

In the title compound, C_10_H_5_IO_3_, an iodinated 3-formyl­chromone derivative, the non-H atoms are essentially coplanar (r.m.s. deviation = 0.0259 Å), with the largest deviation from the least-squares plane [0.056 (5) Å] being found for the formyl O atom. In the crystal, mol­ecules are linked through I⋯O halogen bonds [I⋯O = 3.245 (4) Å, C—I⋯O = 165.95 (13) and C=O⋯I = 169.7 (4)°] along [101]. The supra­molecular chains are assembled into layers *via* π–π stacking inter­actions along the *b* axis [shortest centroid–centroid distance between the pyran and benzene rings = 3.558 (3) Å].

## Related literature   

For related structures, see: Ishikawa (2014*a* ▶,*b* ▶,*c* ▶). For the synthesis of the precursor of the title compound, see: Bovonsombat *et al.* (2009 ▶). For halogen bonding, see: Auffinger *et al.* (2004 ▶); Metrangolo *et al.* (2005 ▶); Wilcken *et al.* (2013 ▶); Sirimulla *et al.* (2013 ▶).
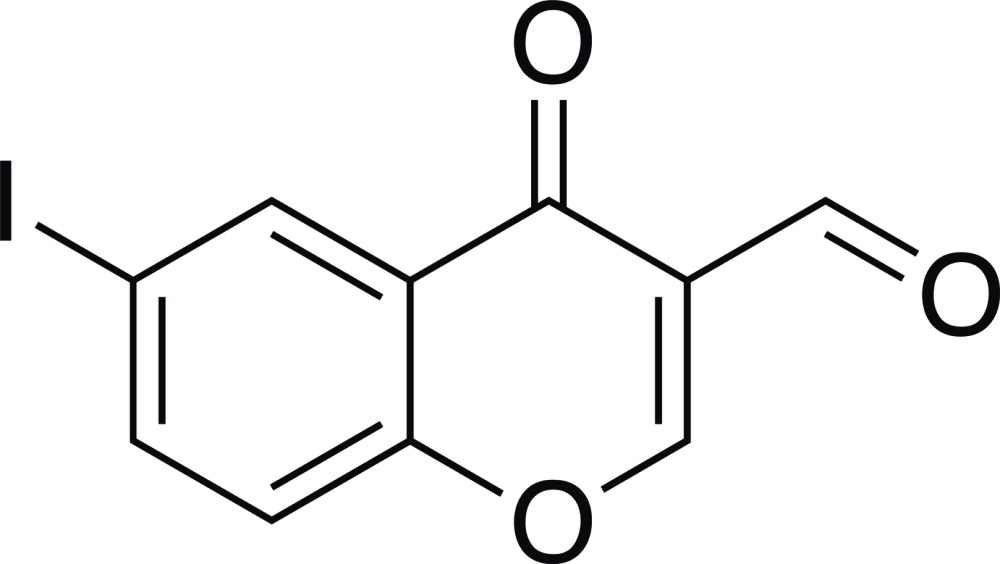


## Experimental   

### 

#### Crystal data  

C_10_H_5_IO_3_
*M* *_r_* = 300.05
Triclinic, 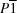

*a* = 6.5741 (17) Å
*b* = 6.798 (3) Å
*c* = 10.437 (5) Å
α = 79.03 (3)°
β = 86.45 (3)°
γ = 76.00 (3)°
*V* = 444.3 (3) Å^3^
*Z* = 2
Mo *K*α radiation
μ = 3.58 mm^−1^
*T* = 100 K
0.25 × 0.25 × 0.08 mm

#### Data collection  

Rigaku AFC-7R diffractometer
Absorption correction: ψ scan (North *et al.*, 1968 ▶) *T*_min_ = 0.432, *T*_max_ = 0.751
2519 measured reflections
2050 independent reflections
1989 reflections with *F* ^2^ > 2σ(*F* ^2^)
*R*_int_ = 0.014
3 standard reflections every 150 reflections intensity decay: −1.8%

#### Refinement  

*R*[*F* ^2^ > 2σ(*F* ^2^)] = 0.039
*wR*(*F* ^2^) = 0.104
*S* = 1.11
2050 reflections
127 parameters
H-atom parameters constrained
Δρ_max_ = 2.55 e Å^−3^
Δρ_min_ = −3.59 e Å^−3^

### 

Data collection: *WinAFC Diffractometer Control Software* (Rigaku, 1999 ▶); cell refinement: *WinAFC Diffractometer Control Software*; data reduction: *WinAFC Diffractometer Control Software*; program(s) used to solve structure: *SHELXS97* (Sheldrick, 2008 ▶); program(s) used to refine structure: *SHELXL97* (Sheldrick, 2008 ▶); molecular graphics: *CrystalStructure* (Rigaku, 2010 ▶); software used to prepare material for publication: *CrystalStructure*.

## Supplementary Material

Crystal structure: contains datablock(s) General, I. DOI: 10.1107/S1600536814012471/tk5319sup1.cif

Structure factors: contains datablock(s) I. DOI: 10.1107/S1600536814012471/tk5319Isup2.hkl

Supporting information file. DOI: 10.1107/S1600536814012471/tk5319Isup3.cml

CCDC reference: 1005730

Additional supporting information:  crystallographic information; 3D view; checkCIF report

## Figures and Tables

**Fig. 1.**
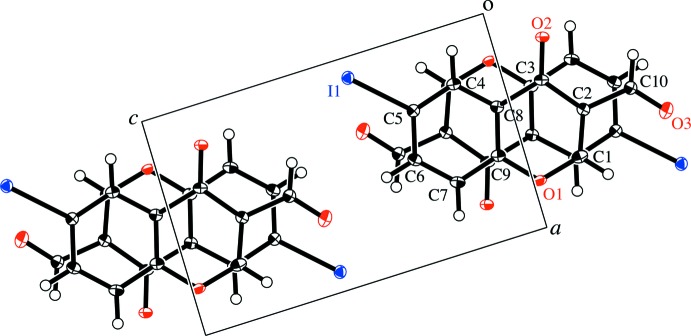
A packing view of the title compound with displacement ellipsoids drawn at the 50% probability level. Hydrogen atoms are shown as small spheres of arbitrary radius.

**Fig. 2.**
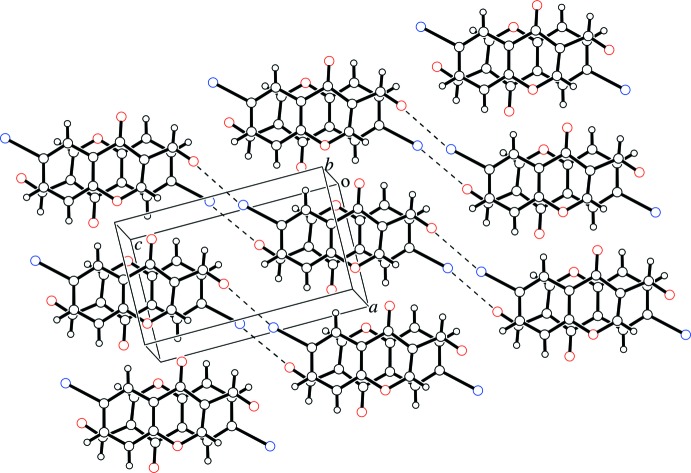
A packing view of the title compound. The intermolecular halogen bonds are represented as dashed lines for I···O.

**Fig. 3.**
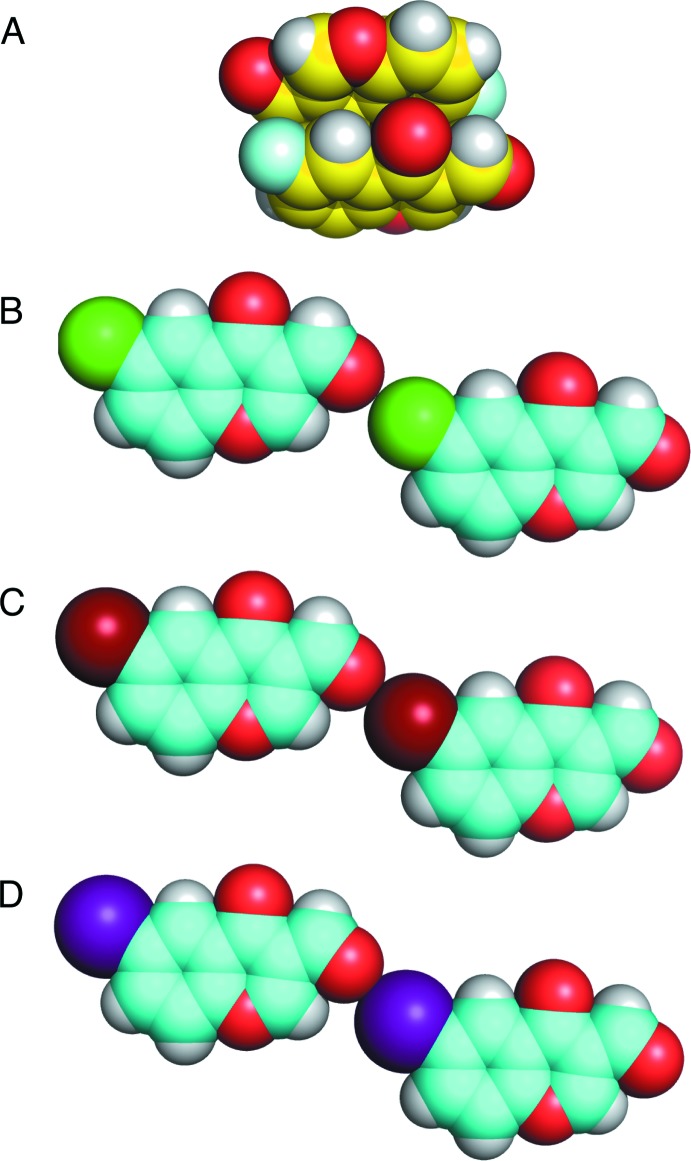
Sphere models of the supramolecular aggregation in the crystal structures of 6-fluoro-4-oxo-4H-chromene-3-carbaldehyde (A), 6-chloro-4-oxo-4H-chromene-3-carbaldehyde (B), 6-bromo-4-oxo-4H-chromene-3-carbaldehyde (C), and the title compound (D).

## supplementary crystallographic information

### S1. Structural commentary

Halogen bonds have been found to occur in organic, inorganic, and biological systems, and have recently attracted much attention in medicinal chemistry, chemical biology and supra­molecular chemistry (Auffinger *et al.*, 2004, Metrangolo *et al.*, 2005, Wilcken *et al.*, 2013, Sirimulla *et al.*, 2013). We have recently reported the crystal structures of monohalogenated 3-formyl­chromone derivatives 6-fluoro-4-oxo-4*H*-chromene-3-carbaldehyde (Ishikawa, 2014*c*, Fig.·3A), 6-chloro-4-oxo-4*H*-chromene-3-carbaldehyde (Ishikawa, 2014*a*, Fig.·3B), and 6-bromo-4-oxo-4*H*-chromene-3-carbaldehyde (Ishikawa, 2014*b*, Fig.·3C). It was found that halogen bond is formed between the formyl oxygen atom and the bromine atom in the bromo derivative, but is not formed in the others light-atom derivatives. As part of our inter­est in this type of chemical bonding, we herein report the crystal structure of a monoiodinated 3-formyl­chromone derivative 6-iodo-4-oxo-4*H*-chromene-3-carbaldehyde. The objective of this study is to reveal whether halogen bond(*s*) can be formed in the crystal structure of the title compound with the iodine atom in the 6-position.

The mean deviation of the least-squares plane for the non-hydrogen atoms is 0.0259 Å, and the largest deviation is 0.056 (5) Å for C10. These mean that these atoms are essentially coplanar (Fig. 1).

In the crystal, the molecules are stacked with the inversion-symmetry equivalents along the *b* axis [shortest centroid–centroid distance between the pyran and benzene^i^ rings of the 4*H*-chromene units = 3.588 (3) Å, *i*: -*x* + 1, -*y* + 2, -*z*], as shown in Fig. 1.

Halogen bond is observed between the iodine atom and the formyl oxygen atom of the translation-symmetry equivalent^ii^ [I1···O3^ii^ = 3.245 (4) Å, *ii*: *x* - 1, *y*, *z* + 1] along [101], as shown in Fig. 2. The angles of C–I···O and I···O=C are 165.95 (13) and 169.7 (4)°, respectively. Thus, it is found that halogen bond is formed for the iodine atom at 6-position, as shown in Fig.·3D. The space group and crystal packing mode of the title compound are the same with those of 6-chloro-4-oxo-4*H*-chromene-3-carbaldehyde and 6-bromo-4-oxo-4*H*-chromene-3-carbaldehyde. On the other hand, halogen bonding is observed for 6-bromo-4-oxo-4*H*-chromene-3-carbaldehyde (Fig.·3C) and the title compound (Fig.·3D), but is not observed for 6-chloro-4-oxo-4*H*-chromene-3-carbaldehyde (Fig.·3B). These should be accounted for by the larger size of the σ holes of the bromine and iodine atoms at 6-position (Wilcken *et al.*, 2013).

### S2. Synthesis and crystallization

2'-Hy­droxy-5'-iodo­aceto­phenone was prepared according to the literature method (Bovonsombat *et al.*, 2009). To a solution of 2'-hy­droxy-5'-iodo­aceto­phenone (1.4 mmol) in *N*,*N*-di­methyl­formamide (5 ml) was added dropwise POCl_3_ (3.4 mmol) for 3 min at 0 °C. After the mixture was stirred for 17 h at room temperature, water (30 ml) was added. The precipitates were collected, washed with water, and dried *in vacuo* (yield: 83%). ^1^H NMR (400 MHz, DMSO-*d*_6_): *δ* = 7.60 (d, 1H, *J* = 8.8 Hz), 8.18 (dd, 1H, *J* = 2.4 and 8.8 Hz), 8.37 (d, 1H, *J* = 2.4 Hz), 8.95 (s, 1H), 10.10 (s, 1H). DART-MS calcd for [C_10_H_5_I_1_O_3_ + H^+^]: 300.936, found 300.947. Single crystals suitable for X-ray diffraction were obtained by slow evaporation of a chloro­form solution of the title compound held at room temperature.

### S3. Refinement

The C(*sp*^2^)-bound hydrogen atoms were placed in geometrical positions [C–H = 0.95 Å, *U*_iso_(H) = 1.2*U*_eq_(C)], and refined using a riding model.

### Crystal data

**Table:**

C_10_H_5_IO_3_	*Z* = 2
*M**_r_* = 300.05	*F*(000) = 284.00
Triclinic, *P*1	*D*_x_ = 2.243 Mg m^−^^3^
Hall symbol: -P 1	Mo *K*α radiation, λ = 0.71069 Å
*a* = 6.5741 (17) Å	Cell parameters from 25 reflections
*b* = 6.798 (3) Å	θ = 15.1–17.0°
*c* = 10.437 (5) Å	µ = 3.58 mm^−^^1^
α = 79.03 (3)°	*T* = 100 K
β = 86.45 (3)°	Plate, yellow
γ = 76.00 (3)°	0.25 × 0.25 × 0.08 mm
*V* = 444.3 (3) Å^3^	

### Data collection

**Table:**

Rigaku AFC-7R diffractometer	*R*_int_ = 0.014
ω–2θ scans	θ_max_ = 27.5°
Absorption correction: ψ scan (North *et al.*, 1968)	*h* = −4→8
*T*_min_ = 0.432, *T*_max_ = 0.751	*k* = −8→8
2519 measured reflections	*l* = −13→13
2050 independent reflections	3 standard reflections every 150 reflections
1989 reflections with *F*^2^ > 2σ(*F*^2^)	intensity decay: −1.8%

### Refinement

**Table:**

Refinement on *F*^2^	Secondary atom site location: difference Fourier map
*R*[*F*^2^ > 2σ(*F*^2^)] = 0.039	Hydrogen site location: inferred from neighbouring sites
*wR*(*F*^2^) = 0.104	H-atom parameters constrained
*S* = 1.11	*w* = 1/[σ^2^(*F*_o_^2^) + (0.0828*P*)^2^ + 0.5762*P*] where *P* = (*F*_o_^2^ + 2*F*_c_^2^)/3
2050 reflections	(Δ/σ)_max_ < 0.001
127 parameters	Δρ_max_ = 2.55 e Å^−^^3^
0 restraints	Δρ_min_ = −3.59 e Å^−^^3^
Primary atom site location: structure-invariant direct methods	

### Special details

**Table:**

Refinement. Refinement was performed using all reflections. The weighted *R*-factor (*wR*) and goodness of fit (*S*) are based on *F*^2^. *R*-factor (gt) are based on *F*. The threshold expression of *F*^2^ > 2.0 σ(*F*^2^) is used only for calculating *R*-factor (gt).

### Fractional atomic coordinates and isotropic or equivalent isotropic displacement parameters (Å^2^)

**Table:**

	*x*	*y*	*z*	*U*_iso_*/*U*_eq_	
I1	0.09130 (3)	0.75924 (3)	0.412452 (19)	0.01417 (14)	
O1	0.7896 (5)	0.6863 (5)	−0.0188 (3)	0.0146 (6)	
O2	0.1807 (5)	0.8519 (5)	−0.1406 (3)	0.0160 (6)	
O3	0.6616 (5)	0.7995 (5)	−0.4137 (3)	0.0204 (7)	
C1	0.7417 (7)	0.7311 (6)	−0.1454 (5)	0.0155 (8)	
C2	0.5449 (6)	0.7856 (6)	−0.1926 (4)	0.0112 (7)	
C3	0.3623 (7)	0.8053 (6)	−0.1040 (4)	0.0112 (7)	
C4	0.2610 (6)	0.7778 (6)	0.1343 (4)	0.0120 (7)	
C5	0.3206 (6)	0.7330 (6)	0.2634 (4)	0.0115 (7)	
C6	0.5314 (7)	0.6711 (6)	0.2983 (5)	0.0136 (8)	
C7	0.6858 (7)	0.6540 (6)	0.2022 (4)	0.0143 (8)	
C8	0.4172 (6)	0.7620 (5)	0.0363 (4)	0.0103 (7)	
C9	0.6270 (7)	0.7003 (6)	0.0722 (4)	0.0126 (8)	
C10	0.5169 (7)	0.8281 (6)	−0.3362 (4)	0.0139 (8)	
H1	0.8547	0.7242	−0.2070	0.0186*	
H2	0.1169	0.8186	0.1124	0.0144*	
H3	0.5684	0.6409	0.3877	0.0163*	
H4	0.8297	0.6115	0.2246	0.0172*	
H5	0.3788	0.8801	−0.3689	0.0167*	

### Atomic displacement parameters (Å^2^)

**Table:**

	*U*^11^	*U*^22^	*U*^33^	*U*^12^	*U*^13^	*U*^23^
I1	0.01465 (19)	0.01545 (19)	0.01309 (19)	−0.00418 (12)	0.00051 (11)	−0.00369 (11)
O1	0.0083 (12)	0.0180 (14)	0.0178 (14)	−0.0035 (10)	−0.0002 (11)	−0.0035 (11)
O2	0.0100 (13)	0.0212 (14)	0.0155 (13)	−0.0030 (11)	−0.0019 (10)	−0.0004 (11)
O3	0.0206 (15)	0.0224 (15)	0.0188 (15)	−0.0056 (12)	0.0051 (12)	−0.0062 (12)
C1	0.0130 (17)	0.0118 (17)	0.023 (2)	−0.0053 (14)	0.0004 (15)	−0.0038 (15)
C2	0.0137 (17)	0.0075 (16)	0.0139 (18)	−0.0043 (13)	−0.0011 (14)	−0.0025 (13)
C3	0.0104 (17)	0.0062 (15)	0.0172 (19)	−0.0024 (13)	−0.0024 (14)	−0.0015 (13)
C4	0.0117 (17)	0.0085 (16)	0.0167 (18)	−0.0032 (13)	−0.0010 (14)	−0.0033 (13)
C5	0.0136 (17)	0.0076 (15)	0.0139 (17)	−0.0041 (13)	0.0002 (13)	−0.0015 (12)
C6	0.0143 (19)	0.0115 (17)	0.0156 (18)	−0.0038 (14)	−0.0032 (15)	−0.0018 (14)
C7	0.0120 (17)	0.0129 (17)	0.0185 (19)	−0.0025 (14)	−0.0035 (14)	−0.0034 (14)
C8	0.0129 (17)	0.0034 (14)	0.0146 (19)	−0.0027 (12)	−0.0017 (14)	−0.0001 (12)
C9	0.0124 (17)	0.0097 (16)	0.017 (2)	−0.0037 (13)	−0.0016 (15)	−0.0042 (14)
C10	0.0145 (17)	0.0130 (17)	0.0144 (19)	−0.0038 (14)	0.0003 (14)	−0.0023 (14)

### Geometric parameters (Å, º)

**Table:**

I1—C5	2.100 (4)	C4—C8	1.404 (6)
O1—C1	1.338 (6)	C5—C6	1.397 (6)
O1—C9	1.383 (5)	C6—C7	1.383 (6)
O2—C3	1.224 (5)	C7—C9	1.390 (6)
O3—C10	1.213 (5)	C8—C9	1.394 (6)
C1—C2	1.353 (6)	C1—H1	0.950
C2—C3	1.466 (6)	C4—H2	0.950
C2—C10	1.485 (6)	C6—H3	0.950
C3—C8	1.487 (6)	C7—H4	0.950
C4—C5	1.383 (6)	C10—H5	0.950
			
O1···C3	2.876 (5)	I1···H4^v^	3.1327
O2···C1	3.579 (5)	I1···H5^xi^	3.3845
O2···C4	2.877 (6)	I1···H5^vi^	3.4429
O2···C10	2.910 (5)	O1···H2^iii^	3.0137
O3···C1	2.811 (6)	O1···H2^iv^	3.5123
C1···C7	3.575 (7)	O2···H1^v^	2.6723
C1···C8	2.762 (6)	O2···H2^vi^	2.6440
C2···C9	2.774 (6)	O2···H4^vii^	3.4433
C4···C7	2.809 (6)	O2···H4^iv^	3.5709
C5···C9	2.746 (6)	O3···H3^x^	2.6765
C6···C8	2.795 (6)	O3···H5^ix^	2.8065
I1···O3^i^	3.245 (4)	C1···H2^vii^	3.5802
O1···O1^ii^	3.254 (4)	C1···H2^iv^	3.4884
O1···O2^iii^	3.154 (5)	C1···H4^ii^	3.3639
O1···C4^iv^	3.554 (5)	C3···H2^vi^	3.5381
O2···O1^v^	3.154 (5)	C4···H1^iv^	3.5034
O2···C1^v^	3.192 (6)	C4···H4^v^	3.3197
O2···C4^vi^	3.358 (5)	C5···H1^iv^	3.5345
O2···C7^vii^	3.510 (6)	C5···H4^v^	3.5834
O3···I1^viii^	3.245 (4)	C6···H5^vii^	3.5870
O3···O3^ix^	3.316 (5)	C6···H5^iv^	3.4606
O3···C6^x^	3.494 (6)	C7···H1^ii^	3.4570
O3···C10^ix^	3.321 (5)	C7···H2^iii^	3.3116
C1···O2^iii^	3.192 (6)	C9···H2^iii^	3.5699
C1···C4^vii^	3.444 (6)	C10···H3^x^	3.3327
C1···C4^iv^	3.358 (6)	C10···H3^vii^	3.5102
C1···C5^iv^	3.548 (6)	C10···H3^iv^	3.4573
C2···C5^iv^	3.526 (6)	C10···H5^ix^	3.4573
C2···C6^iv^	3.568 (6)	H1···O2^iii^	2.6723
C3···C7^vii^	3.555 (7)	H1···C4^iv^	3.5034
C3···C7^iv^	3.564 (6)	H1···C5^iv^	3.5345
C3···C9^vii^	3.373 (6)	H1···C7^ii^	3.4570
C3···C9^iv^	3.457 (7)	H1···H2^vii^	3.5954
C4···O1^iv^	3.554 (5)	H1···H2^iv^	3.4845
C4···O2^vi^	3.358 (5)	H1···H4^ii^	2.7142
C4···C1^vii^	3.444 (6)	H2···O1^v^	3.0137
C4···C1^iv^	3.358 (6)	H2···O1^iv^	3.5123
C5···C1^iv^	3.548 (6)	H2···O2^vi^	2.6440
C5···C2^iv^	3.526 (6)	H2···C1^vii^	3.5802
C6···O3^xi^	3.494 (6)	H2···C1^iv^	3.4884
C6···C2^iv^	3.568 (6)	H2···C3^vi^	3.5381
C6···C10^vii^	3.430 (7)	H2···C7^v^	3.3116
C6···C10^iv^	3.438 (7)	H2···C9^v^	3.5699
C7···O2^vii^	3.510 (6)	H2···H1^vii^	3.5954
C7···C3^vii^	3.555 (7)	H2···H1^iv^	3.4845
C7···C3^iv^	3.564 (6)	H2···H2^vi^	3.2186
C8···C8^iv^	3.591 (6)	H2···H4^v^	2.7025
C8···C9^iv^	3.561 (6)	H3···I1^xii^	3.4972
C9···C3^vii^	3.373 (6)	H3···O3^xi^	2.6765
C9···C3^iv^	3.457 (7)	H3···C10^xi^	3.3327
C9···C8^iv^	3.561 (6)	H3···C10^vii^	3.5102
C10···O3^ix^	3.321 (5)	H3···C10^iv^	3.4573
C10···C6^vii^	3.430 (7)	H3···H3^xii^	2.9731
C10···C6^iv^	3.438 (7)	H3···H5^xi^	3.2942
I1···H2	3.0799	H3···H5^vii^	3.5164
I1···H3	3.0514	H3···H5^iv^	3.3247
O1···H4	2.5116	H4···I1^iii^	3.1327
O2···H2	2.6234	H4···O2^vii^	3.4433
O2···H5	2.6408	H4···O2^iv^	3.5709
O3···H1	2.4759	H4···C1^ii^	3.3639
C1···H5	3.2831	H4···C4^iii^	3.3197
C3···H1	3.2973	H4···C5^iii^	3.5834
C3···H2	2.6956	H4···H1^ii^	2.7142
C3···H5	2.7153	H4···H2^iii^	2.7025
C4···H3	3.2803	H5···I1^x^	3.3845
C5···H4	3.2663	H5···I1^vi^	3.4429
C6···H2	3.2850	H5···O3^ix^	2.8065
C8···H4	3.2886	H5···C6^vii^	3.5870
C9···H1	3.1874	H5···C6^iv^	3.4606
C9···H2	3.2740	H5···C10^ix^	3.4573
C9···H3	3.2505	H5···H3^x^	3.2942
C10···H1	2.5492	H5···H3^vii^	3.5164
H1···H5	3.4835	H5···H3^iv^	3.3247
H3···H4	2.3427	H5···H5^ix^	3.4207
I1···H3^xii^	3.4972		
			
C1—O1—C9	118.2 (4)	C4—C8—C9	119.0 (4)
O1—C1—C2	125.1 (4)	O1—C9—C7	115.8 (4)
C1—C2—C3	120.7 (4)	O1—C9—C8	122.3 (4)
C1—C2—C10	118.9 (4)	C7—C9—C8	121.9 (4)
C3—C2—C10	120.4 (4)	O3—C10—C2	123.2 (4)
O2—C3—C2	123.7 (4)	O1—C1—H1	117.453
O2—C3—C8	122.5 (4)	C2—C1—H1	117.444
C2—C3—C8	113.7 (4)	C5—C4—H2	120.591
C5—C4—C8	118.8 (4)	C8—C4—H2	120.596
I1—C5—C4	119.9 (3)	C5—C6—H3	120.161
I1—C5—C6	118.4 (3)	C7—C6—H3	120.160
C4—C5—C6	121.7 (4)	C6—C7—H4	120.551
C5—C6—C7	119.7 (4)	C9—C7—H4	120.551
C6—C7—C9	118.9 (4)	O3—C10—H5	118.395
C3—C8—C4	121.2 (4)	C2—C10—H5	118.392
C3—C8—C9	119.8 (4)		
			
C1—O1—C9—C7	−179.3 (4)	C8—C4—C5—I1	179.1 (3)
C1—O1—C9—C8	−1.0 (6)	C8—C4—C5—C6	−0.5 (6)
C9—O1—C1—C2	−1.5 (6)	H2—C4—C5—I1	−0.9
C9—O1—C1—H1	178.5	H2—C4—C5—C6	179.5
O1—C1—C2—C3	1.7 (6)	H2—C4—C8—C3	−0.9
O1—C1—C2—C10	−179.1 (4)	H2—C4—C8—C9	−179.5
H1—C1—C2—C3	−178.3	I1—C5—C6—C7	−179.5 (3)
H1—C1—C2—C10	0.9	I1—C5—C6—H3	0.5
C1—C2—C3—O2	−179.2 (4)	C4—C5—C6—C7	0.1 (6)
C1—C2—C3—C8	0.4 (6)	C4—C5—C6—H3	−179.9
C1—C2—C10—O3	6.5 (6)	C5—C6—C7—C9	0.3 (6)
C1—C2—C10—H5	−173.5	C5—C6—C7—H4	−179.7
C3—C2—C10—O3	−174.3 (4)	H3—C6—C7—C9	−179.7
C3—C2—C10—H5	5.7	H3—C6—C7—H4	0.3
C10—C2—C3—O2	1.6 (6)	C6—C7—C9—O1	178.0 (4)
C10—C2—C3—C8	−178.7 (3)	C6—C7—C9—C8	−0.4 (7)
O2—C3—C8—C4	−1.6 (6)	H4—C7—C9—O1	−2.0
O2—C3—C8—C9	177.0 (4)	H4—C7—C9—C8	179.7
C2—C3—C8—C4	178.7 (3)	C3—C8—C9—O1	3.1 (6)
C2—C3—C8—C9	−2.7 (5)	C3—C8—C9—C7	−178.7 (4)
C5—C4—C8—C3	179.1 (3)	C4—C8—C9—O1	−178.3 (4)
C5—C4—C8—C9	0.5 (6)	C4—C8—C9—C7	−0.1 (6)

Symmetry codes: (i) *x*−1, *y*, *z*+1; (ii) −*x*+2, −*y*+1, −*z*; (iii) *x*+1, *y*, *z*; (iv) −*x*+1, −*y*+2, −*z*; (v) *x*−1, *y*, *z*; (vi) −*x*, −*y*+2, −*z*; (vii) −*x*+1, −*y*+1, −*z*; (viii) *x*+1, *y*, *z*−1; (ix) −*x*+1, −*y*+2, −*z*−1; (x) *x*, *y*, *z*−1; (xi) *x*, *y*, *z*+1; (xii) −*x*+1, −*y*+1, −*z*+1.
